# Can learnings from the COVID-19 pandemic improve trial conduct post-pandemic? A case study of strategies used by the DISC trial

**DOI:** 10.1177/26320843221128296

**Published:** 2022-09-22

**Authors:** Catherine Knowlson, Puvan Tharmanathan, Catherine Arundel, Sophie James, Lydia Flett, Samantha Gascoyne, Charlie Welch, David Warwick, Joseph Dias

**Affiliations:** 1 Department of Health Sciences, York Trials Unit, York, UK; 2 University Hospital Southampton, York, UK; 3 University Hospitals of Leicester NHS Trust, York, UK

**Keywords:** Randomised controlled trial, trial management, COVID-19, pandemic, recruitment, retention, intervention delivery, dupuytren’s

## Abstract

**Background:**

RCTs often face issues such as slow recruitment, poor intervention adherence and high attrition, however the 2020/2021 COVID-19 pandemic intensified these challenges. Strategies employed by the DISC trial to overcome pandemic-related barriers to recruitment, treatment delivery and retention may be useful to help overcome routine problems.

**Methods:**

A structured survey and teleconference with sites was undertaken. Key performance indicators in relation to recruitment, treatment delivery and retention were compared descriptively before and after the pandemic started. This was situated also in relation to qualitative opinions of research staff.

**Results:**

Prior to the pandemic, retention was 93.6%. Increased support from the central trial management team and remote data collection methods kept retention rates high at 81.2% in the first 6 months of the pandemic, rising to 89.8% in the subsequent 6 months. Advertising the study to patients resulted in 12.8 patients/month enquiring about participation, however only six were referred to recruiting sites. Sites reported increased support from junior doctors resolved research nurse capacity issues. One site avoided long delays by using theatre space in a private hospital.

**Conclusions:**

Recruitment post-pandemic could be improved by identification of barriers, increased support from junior doctors through the NIHR associate PI scheme and advertising. Remote back-up options for data collection can keep retention high while reducing patient and site burden. To future proof studies against similar disruptions and provide more flexibility for participants, we recommend that RCTs have a back-up option of remote recruitment, a back-up location for surgeries and flexible approaches to collecting data.

## Introduction

There are a number of barriers to health research that were present before the COVID-19 pandemic and will likely remain once we reach a “new normal.” Recruitment and retention are two key areas underpinning the success of a randomised controlled trial (RCT). Failure to obtain outcome data from the predetermined sample size results in under-powered statistical tests, imprecise treatment effect estimates and research waste.^1–3^ Accordingly, research into methods to enhance RCT recruitment and retention are two of the main priorities for clinical trial units.^4^

In 2017, a study by Walters *et al.* identified that around one in 5 RCTs funded by the National Institute for Health Research’s Health Technology Assessment programme fail to reach 80% of the target number of participants.^5^ Even if a RCT recruits to target, this does not guarantee that the desired statistical power will be reached as the target is based upon a predicted attrition rate.^6^ Walters *et al.* identified an average retention rate across HTA–funded RCTs of 89%, however this ranged from 23 to 100% across included studies.^5^ Loss of more than 20% of participants is considered a major threat to the trial validity.^7^

The validity of the RCT findings could also be affected by problems with intervention delivery e.g. poor medication adherence or participant cross-overs.^8,9^ This is handled by estimating the effect of allocation to the treatments under study using intention-to-treat analyses, so even if a participant has crossed over or not adhered to the specified intervention they will be analysed in the group to which they were randomised.^9, 10^ However, if a substantial number of participants have not received their allocated treatment, this can cause estimates of treatment efficacy to be biased (in either direction depending on the treatments being compared and patterns of non-adherence observed).^9,10^

On top of the standard recruitment, adherence and retention barriers, the COVID-19 pandemic brought unprecedented challenges for the delivery of health research in 2020–2021.^11^ Although urgent public health studies for COVID-19 treatments and vaccines were successful in setting up and recruiting participants promptly, the prioritisation of these crucial and urgent COVID-19 studies meant that other ongoing studies became lower priority. The UK’s National Institute for Health Research (NIHR) called for the majority of its non-COVID-19 studies to pause activity, while some studies closed early as completion was no longer feasible.^12–14^ The US responded similarly, as their National Institute of Health reported that around 80% of non-COVID-19 RCTs temporarily or permanently stopped.^15^

Termination of RCTs results in research waste due to the substantial amount of time, effort and funding invested.^16^ Trial management teams worked hard to avoid study closures, using innovative methods to overcome the challenges posed by the COVID-19 pandemic and to adapt processes to maintain participant safety and avoid unrecoverable loss of data.^11^ From this experience, researchers will be better prepared to deal with a similar disruption in the future should it happen again. We may also be able to apply what we have learned from this experience to mitigate routine challenges to RCT conduct.

## Case study: The DISC trial

This paper focuses on the learnings from a single RCT of elective interventions during the COVID-19 pandemic, the Dupuytren’s Interventions Surgery vs. Collagenase (DISC) trial (ISRCTN18254597),^17^ to consider whether strategies employed during this time could help tackle routine challenges. As a study where one of the arms has to be delivered in theatre but the other can be done in clinic, this RCT can provide a unique insight into issues caused by the COVID-19 pandemic and solutions to overcome these, which may be relevant to streamlining future research design and implementation.

The aim of the DISC non-inferiority multi-centre RCT is to compare the effectiveness and cost-effectiveness of two treatment options for Dupuytren’s Contracture: surgical intervention (limited fasciectomy) and injection of an enzyme (collagenase).^17^ The primary outcome is a patient reported outcome on hand function, collected by questionnaire.^17^ The key secondary outcome is recurrence of the contracture, which pre-pandemic required assessment of the hand in clinic.^17^

The DISC trial was in the 30th month of an intended 36 month recruitment period, and on track to reach the target of 710 participants, when recruitment had to pause. As elective procedures were postponed and face-to-face appointments were not possible during the national lockdown, study processes were adapted to allow trial activity to continue throughout the pandemic. The challenges faced and the strategies implemented to try and overcome these are discussed for three key areas: recruitment, treatment delivery and retention.

## Aims

This structured evaluation aims to investigate the strategies implemented during the pandemic to address challenges with recruitment, treatment delay and retention for the DISC trial. The learnings will offer potential strategies which future RCTs should consider incorporating when in the design phase, to assess whether they can overcome routine challenges around recruitment and data collection and to incorporate into contingency plans for future healthcare crises, so that non-urgent public health research can continue with minimal disruption.

## Methods

### Overview

The DISC trial and all changes made to adapt the protocol during the COVID-19 pandemic were approved by the Ethics Committee (Leeds West REC reference: 17/YH/0120) and Health Research Authority (IRAS ID: 208838). All participants provided written informed consent before taking part.

A structured evaluation was conducted to investigate whether these strategies were effective for DISC and whether they could be useful methods for future RCTs to adopt in their contingency plans and to evaluate their effectiveness outside of a pandemic. The descriptive evaluation approach involved:

1. Identification of key elements to compare performance before and after the COVID-19 pause.
2. Checking for routinely collected study data, including process data, which could be used to determine the necessary summary statistics.
3. Supplement with quantitative survey data and opinions of participating sites collected during a teleconference.
  • The site survey was generated on Google Forms, issued by email to all site research teams on 10^th^ November 2020, and analysed using Microsoft Excel. Preliminary results were discussed with the clinical teams at a video conference to provide a qualitative aspect to supplement these findings. Due to the limited data available, thematic analysis was not possible.

**Figure 1.**
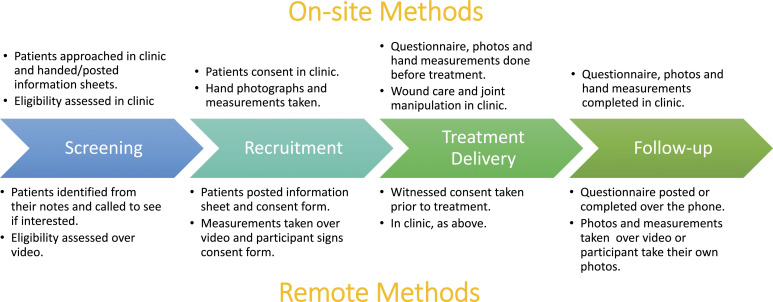
DISC patient pathway including on-site and remote options for screening, recruitment and data collection at all time-points. Treatment delivery always took place in clinic (theatre for the surgery arm).

The strategies employed for each trial process and the key performance indicators were as follows:

a) Recruitment: Three strategies were implemented to address substantially reduced patient referrals once the trial sponsor issued approval to recommence recruitment on 29^th^ June 2020: sites were encouraged to review outstanding referrals received before the pause; General Practitioners (GPs) were encouraged to refer Dupuytren’s patients to the nearest participating hospital, and the British Dupuytren’s Society were contacted to advertise the study through social media. In anticipation of patient concern about attending hospital appointments, a remote pathway was developed as an option for recruitment (Figure 1).
  • The impact of these strategies on recruitment was determined by the screening and recruitment rates. Screening rates and reasons for ineligibility and non-consent were obtained from screening forms routinely returned by sites (most recent data collected 9^th^ August 2021). Recruitment rates were calculated based on the number of randomisations per month pre-pause (from the start of the recruitment period to the recruitment pause) and post-pause (from the date of approval to unpause to 31^st^ August 2021).
b) Treatment delivery: Sites were urged to book treatment appointments as soon as possible. As the follow-up time points correspond to the treatment delivery date, it was important to have these completed promptly so that primary outcome data (1 year after treatment) could be collected within the funded period. In addition, the progressive nature of Dupuytren’s disease meant patients’ contractures could potentially worsen to Tubiana grade 4 (extension deficit >135 degrees),^7^ which would render them ineligible for the trial. Sites were reminded of the protocol requirement for taking goniometric measurements of the fingers prior to treatment to ensure ineligible patients did not receive treatment. This also allowed for any changes since baseline to be accounted for in the analysis.
  • An average delay (in terms of the arithmetic mean) was calculated using the number of days between the baseline and treatment delivery appointments for each participant whose treatment delivery CRF had been returned. Where CRFs had not yet been returned, sites were contacted to confirm whether treatment had taken place to monitor the number of patients awaiting treatment.
c) Retention: To ensure that follow-up data could be collected while non-essential clinic appointments were cancelled, remote methods were implemented to collect researcher-reported (telephone or video consultations) and participant-reported (postal or telephone questionnaires) data on investigator and participant Case Report Forms (CRFs), respectively. To remotely collect secondary outcome data relating to recurrence of the contracture, participants were asked to return photographs of their hand. At sites where COVID-19 burden meant that research nurses were redeployed, follow-ups were temporarily supported by the DISC trial management team. Most outcomes were captured via questionnaire, except the key secondary outcome of recurrence could only be collected by video appointment to capture images of the hand or if participants were able to send a photograph.
  • Retention was monitored by assessing the percentage of expected 1 year follow-up participant questionnaires completed, as these contain the primary outcome data, and the number of withdrawals pre- and post-recruitment pause.
  • As new methods of follow-up were introduced during the pandemic, the opinions of the DISC trial management team and clinical teams on these methods were obtained at team meetings and via email/telephone communication, respectively, and summarised as advantages and disadvantages. The method of follow-up used by each site was determined from correspondence.

## Results

### Recruitment

The average number of Dupuytren’s patients screened before the pause was 33.4 patients/month (1^st^ May 2017–19^th^ March 2020). This reduced to 7.3 patients/month from 26^th^ June 2020 (one site restarted 3 days before Sponsor approval) to 30^th^ April 2021. Correspondingly, recruitment decreased from 16.9 patients/month on average to 4.8 patients/month, although the percentage who consented to take part did not change substantially from 74.0% to 76.5%.

In addition to screening of patient lists, the DISC trial was also advertised by the British Dupuytren’s Society through social media from 25^th^ November 2020. Up to 24^th^ May 2021, 77 patients expressed interest (averaging 12.8 patients/month). Patients were advised of the nearest recruiting site to request referral by their GP if appropriate. In total, six patients (7.8%) identified in this way were recruited.

**Figure 2.**
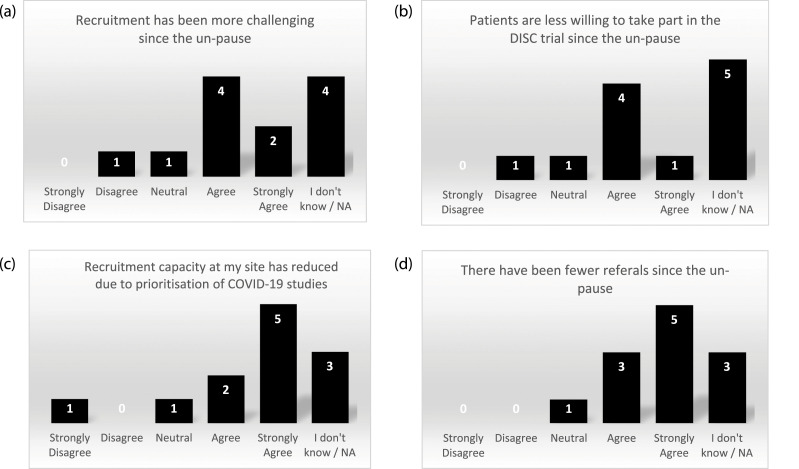
Site survey results for COVID-19 impact on recruitment to the DISC trial.

All 32 sites participating in the DISC trial were sent the site survey on the impact of COVID-19 on the DISC trial. Researchers from12 of the sites completed the survey. Half of the responding sites reported finding recruitment more challenging following un-pause, primarily because patients were less willing to take part (Figure 2(a), and (b)). However, this does not reflect the above rate of conversion from screening to recruitment. Furthermore, for those patients screened, concern over COVID-19 risk was not given as a reason for not taking part. Sites had reduced recruitment capacity due to prioritisation of COVID-19 studies (Figure 2(c)). During discussion around reduced capacity, sites reported staff shortages as “the research team have had a lot of COVID” (RN), and “research nurses have been redeployed to COVID and vaccine studies” (Principal Investigator (PI)). To overcome this issue, one site reported “continuing to recruit with PI and reg [registrar] doing most of the work and research nurse helping when she can” (RN) but another found that “recruiting to non-COVID studies is low on the agenda for a lot of our doctors” (RN). Eight sites reported receiving fewer referrals (Figure 2(d)), with agreement that “people are not willing to come into the hospital for non-urgent problems,” (RN) and “patients are reluctant to come in” (RN).

To overcome the reluctance of patients to attend clinics in person, one site used the remote recruitment pathway to recruit a patient. They shared their experience with other sites: “The patient was very comfortable (with the process). He didn’t want to come in, he was absolutely sure about that… we’re happy about doing it remotely” (research nurse (RN)). The resulting data was still of high quality as all information could be captured by video appointment.

### Treatment delivery

Postponement of elective procedures caused delays for both trial interventions. Prior to the first UK lockdown, the average delay between the baseline appointment and treatment delivery was 79.7 (SD 60.2) days, with a median of 67.0 days (Q1 41.0, Q3 98.0) as calculated on 29^th^ February 2020. The pandemic caused this delay to increase to an average of 99.9 (SD 100.1) days, with a median of 70.0 days (Q1 41.0, Q3 116.0) as of 31^st^ July 2021. As the injection could be delivered in clinic rather than in theatre, the delay was limited for this arm at an average of 80.4 (SD 83.5) days and a median of 56.0 days (Q1 33.0, Q3 87.0) compared to an average of 122.6 (SD 112.4) days and a median of 86.0 days (Q1 58.0, Q3 142.0) in the surgical arm. These values do not account for participants still awaiting treatment, which had cumulated to 64 participants (of 576 recruited prior to the first national lockdown) as of 21st August 2020 (40 control, 24 intervention). This reduced slowly over time to 19 participants awaiting treatment as of 31^st^ July 2021 (14 control, 5 intervention). Two patients had contractures which progressed so far that they were no longer eligible for either trial treatment.

**Figure 3.**
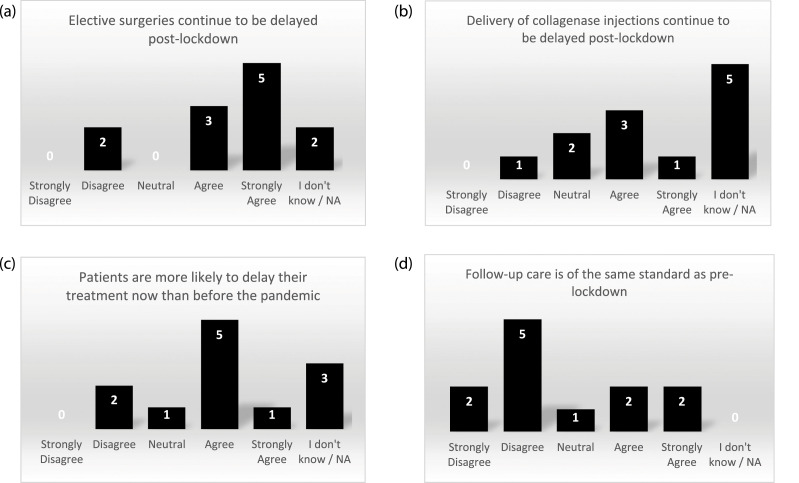
Site survey results on COVID-19 impact on treatment delivery for the DISC trial.

The site survey identified that while elective surgeries and collagenase injections continued to be delayed after lockdown, participants were also choosing to delay their treatment (Figure 3(a)–(c)). Sites reported that other reasons for delays were “a backlog of people on the waiting list” (RN) and “cancellations due to staff shortages” (RN). Some sites shared strategies they used to deliver study treatments: “delivering treatments in the private hospitals” (PI) and “if collagenase is delivered in clinic... (participants) probably feel a little safer than they would do if they had to come into a theatre environment” (research administrator). Given the move to remote appointments, seven responders disagreed that follow-up care post-treatment was of the same standard as pre-lockdown (Figure 3(d)).

### Retention

In the 6 months prior to the recruitment pause, return of primary outcome data (1 year follow-up questionnaires) was at 93.6%. This decreased to 81.2% in the 6 months following the pause, but then increased in the subsequent 6 months (89.8%). There was no increase in withdrawals (11 and 9 participants in the 6 months pre- and post-pause, respectively).

Instruction packs for participants to take photographs of their hand were posted to 51 participants between 7^th^ April 2020 and 2^nd^ November 2020, and 17 participants (33%) returned photographs. One site developed a method of taking goniometric measurements and hand photographs during a video appointment, which was implemented by five sites. The DISC trial management team assisted with follow-ups for 13 of the 32 sites. The DISC trial management team and clinical teams found each of these methods to have distinct advantages and disadvantages (Table 1).

**Table 1.** Evaluation of remote retention strategies.

Change implemented	Advantages	Disadvantages
Postal questionnaires	•All sites able to implement •No technology required •Can be done by central trial management team if needed	•Postal costs
Telephone questionnaires	•Easy to implement •Can be done by central trial management team if needed	•Time consuming •Data could be inaccurate if patients do not want to be honest about personal questions
Telephone follow-ups	•Majority of patients will have access to a telephone •All sites able to implement	•Recurrence can only be assessed if patients send a photograph •Only possible if patients have provided a contact number •Patients aren’t always available when they say they will be
Video follow-ups	•All outcome data can be collected •More personal than telephone	•Not all patients have the technology or the willingness to use this method
Follow-up by the DISC trial management team at york trials unit (YTU)	•Reduces burden on site teams	•Lack of familiarity with the caller •YTU staff are not medically trained so any issues need to be passed on to the clinical team •Efficient communication required between trial unit and site

**Figure 4.**
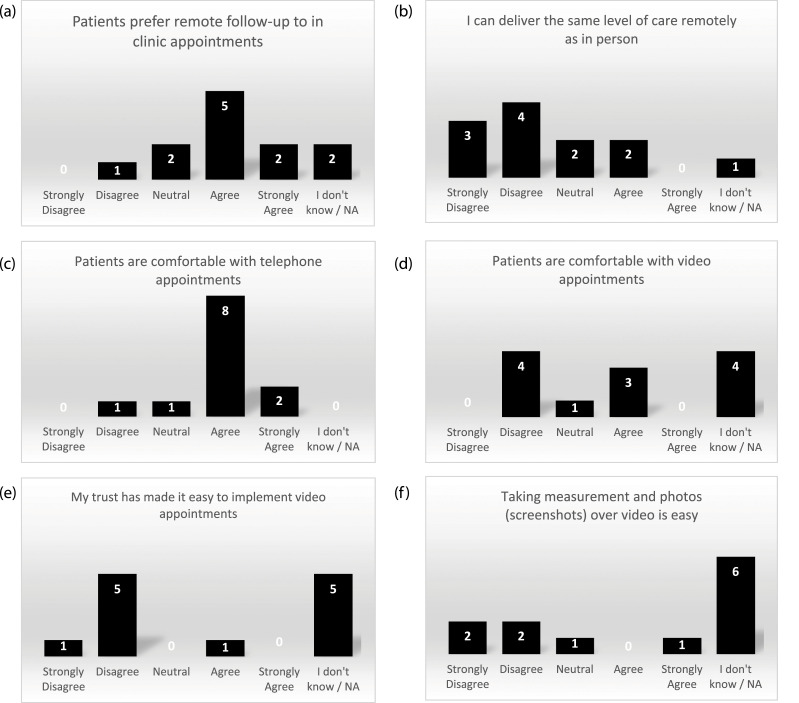
Site survey results on COVID-19 impact on retention for the DISC trial.

In the site survey, seven of the 12 sites agreed that patients preferred remote follow-ups to clinic appointments (Figure 4(a)). At the teleconference, it was agreed that both COVID-19 risk and convenience were reasons for this, with one site stating that “there will be a move to continue with remote appointments if and when we see an end to COVID” (RN). However, seven survey respondents disagreed that the same level of care could be delivered remotely as in person (Figure 4(b)).

Generally, sites felt that patients were comfortable with telephone appointments, but their responses for video appointments varied (Figure 4(c) and (d)). Feedback from one site was that video appointments “seem to be quite acceptable to our patients, but phone will always be more popular with the technophobes” (RN). The site which developed the method of data collection during a video appointment felt that this method “was obviously not as good as seeing patients face to face…we can now see patients face to face so we’re going back to the traditional route” (research practitioner). Another problem with video appointments was that six sites disagreed that their NHS Trust made it easy to implement (Figure 4(e)) and four sites disagreed that taking measurements and photos over video was easy (Figure 4(f)). This was elaborated on by one site which found that “the main issue has been the deployment of cameras in clinic” (PI) and another site where they “have not had an option of video follow up that is reliable” (PI).

## Discussion

The DISC trial is a prime example of a RCT that was faced with unforeseen challenges, but with the strategies employed it was able to continue without excessive loss of data. The challenges presented by the COVID-19 pandemic for the conduct of non-urgent RCTs were all present here. This is in addition to expected barriers to recruitment, intervention adherence and retention. The challenges faced by the study, where both study arms are elective scheduled procedures but one can be delivered during a clinic visit while the other must take place in theatre, provides a unique insight into a range of barriers caused by the COVID-19 pandemic. As the follow-up time points correspond to the treatment delivery date, this provided an additional concern around completed primary outcome data collection at 1 year within the funded period. This investigation of methods to overcome these recruitment, intervention delivery and data collection issues provided guidance for contingency plans and gives ideas for methods that could tackle routine barriers in real-time, although evaluation in a pre-planned study with statistical analyses is required to determine the effectiveness of the strategies across different trials and outside of a pandemic.

Although the methods used by the DISC trial were related to the specific problems arising due to the pandemic, these methods could also be useful to RCTs post-pandemic. There is much interest in research into methods to improve RCT recruitment rates.^18,19^ In the UK, optimising RCT delivery using remote methods is a key piece of work being developed between research funders and regulators.^20^

The DISC survey and site investigators meeting identified a number of problems effectively and is an approach recommended for ongoing/future trials. Communication with study sites to identify what barriers exist so that solutions can be developed is a proven effective strategy to improve recruitment rates.^21,22^ Additionally, there are resources to help RCTs experiencing issues such as the Quintet recruitment intervention to identify and overcome recruitment barriers^18^ and the NIHR Trial Delivery group which offers advice on any challenges impeding timely completion of non-commercial studies.^23^

The use of direct promotion of studies to patients, even when it is not the primary method of recruitment, is a useful strategy for contingency plans as it can generate continued interest from the relevant patient group by making them aware of the study continuing during major upheavals. The DISC trial team had established communication with the important patient support group in the UK, the British Dupuytren’s Society, at the onset of the study. This established relationship was used to enhance direct contact with patients when communication with sites identified that referrals for Dupuytren’s Contracture were much lower than usual after recruitment restarted. This is thought to be because patients delayed visits to their GP for fear of coronavirus exposure or concern over burdening the NHS.^24^ It was evident from the response rates to British Dupuytren’s Society advertisements for DISC that a proportion of Dupuytren’s patients were still seeking treatment, however only a small proportion of these patients were randomised. We suspect this is likely due to delays with GP or hospital appointments due to the pandemic. Although it is not possible to test whether this advertising approach would have been effective for DISC once the healthcare sector recovers (as recruitment has now ended), the sheer response seen when this activity was stepped up underscores the importance of patient awareness and engagement here. Therefore, we still recommend consideration be given to having a broad and flexible approach to study promotion, especially as other studies have shown some success at improving recruitment rates and reducing recruitment costs with well thought through approaches.^25–27^

Another recommended method to enhance recruitment is through having an understanding on how to be flexible in terms of the make-up of study sites and being clear on personnel who might be able to undertake varying tasks with recruitment and retention of participants. In the UK, the NIHR Associate Principal Investigator (API) scheme has been established in order to provide a more expanded approach and future thinking on how study site leadership and oversight can be organised. The API scheme formalises the way to train and upskill research-active personnel at study sites.^28^ This scheme was previously limited to surgical trainees but is now open to a number of specialities.^28^ Some DISC sites reported registrars and trainees supporting research nurse activities during the pandemic due to established relationships. This extends beyond the pandemic and shows the importance of ensuring a more integrated study team at sites is established from the outset.

The DISC remote recruitment pathway was developed specifically for the pandemic as face-to-face clinics were cancelled, and would therefore be a useful strategy for other RCTs to have in their contingency plans should similar disruptions to clinic appointments occur in the future. Unfortunately for DISC, this method was hindered by some trusts not being able to implement video appointments and a particularly low rate of referrals, however it may become more effective in the future if video appointments become more routine. This can be a very effective method of recruitment; for example, one site alone using this approach was able to recruit more patients than 28 sites recruiting face to face.^29^ For studies which do not require a face to face assessment, recruitment by telephone could be utilised to avoid the need for video technology.^30^ In a post-pandemic scenario, there will still be patients unable or unwilling to attend clinic for various reasons and therefore a remote recruitment pathway would avoid missing these potential participants. Additional advantages include reduced recruitment costs and a larger pool of patients who can be approached.^29,31^ The success may be dependent on the type of research as the coordinators of one study which used video recruitment suspected that is may have affected the recruiter’s ability to gain trust from the participant.^32^

While the pandemic was certainly the first major upheaval to affect hospital capacity nationwide, it is not uncommon for individual site teams to experience capacity issues such as staffing shortages. To prepare for such instances, we would recommend planning what can be carried out by the central trial management team should this be required. One of the benefits of remote data collection is to allow the activity of follow-ups to be taken up by non-hospital staff. Additionally this benefits the patients by minimising the time and financial burden of attending hospital appointments, which could improve recruitment and retention.^33^

When the first nationwide lockdown was announced in 2020, the initial changes to data collection by post or telephone were straightforward for the DISC team to implement. The substantial drawback of this method was that key secondary outcome data on recurrence could only be captured if the patient took a photograph of their hand. This was not possible for all patients, possibly due to them lacking the required technology. One of the site PIs (DW) was innovative in taking the burden of patients taking their own photographs and implemented video appointments where screenshots could be captured to take measurements from these. This was a novel strategy that worked effectively at sites which were able to implement it, and would be a great back-up option for other RCTs with data that can only be collected remotely by visual assessment. However, this approach was necessarily limited to patients with the required technology. As methods of data collection each have advantages and disadvantage, it is recommended to have a range of options available to suit both sites and participants. Depending on the care that patients require, remote follow-up methods may only be suitable as a last resort in some RCTs as the medical staff in the DISC trial report a reduction in the quality of care that can be delivered remotely.

Treatment delivery was particularly challenging for DISC during the pandemic as both treatments are elective procedures. From a pragmatic perspective, it could be argued that longer waiting times in the surgery group are an unavoidable component of this treatment as delivered within the NHS (both prior to and during the COVID-19 pandemic), therefore any consequences of this imbalance on outcome should be accepted. We would recommend anticipating potential delays, whether nationwide or site-specific and having pre-treatment data collection and sensitivity analysis conditioning on the primary measure at this time point rather than baseline to account for any changes during the delay for progressive conditions. All surgical RCTs should proactively anticipate imbalances in treatment delivery times and account for this in their analysis plan, as a pre-specified plan is important to prevent bias.^14^

It was difficult to influence sites to schedule treatments any quicker during the pandemic, given there were more urgent priorities; therefore, having a back-up option for delivering treatment in case of unprecedented delays would be useful to contingency plans. The only method that sites found could avoid treatment delays was to make use of an additional location linked to the participating NHS trust such as a private clinic. In other studies, changes were made to supply their intervention to patient homes or change who delivers it,^30^ although this was not feasible for the DISC interventions.

### Recommendations

The DISC trial faced enhanced barriers to recruitment, treatment delivery and retention during the COVID-19 pandemic. As RCTs often face challenges with these elements during routine trial conduct, we would recommend that other trials implement the following methods, and evaluate their effectiveness, as appropriate:

• Advertise the study via social media, on a study website and through relevant societies to reach as many patients as possible and facilitate their referral to trial sites.
• Encourage research-active staff in the NHS to support recruitment and retention activities, and take part in the NIHR associate PI scheme.
• Have a back-up location to deliver treatments should there be unprecedented delays at a site.
• Create back-up plans for study activities and visits usually carried out in clinic or by site staff, which sets out how these will be handled if clinics are cancelled or staff are unavailable:
  ➢ Consider using a “blended” approach between the trials unit and clinical teams to support site activity where they have reduced capacity, for example posting questionnaires or calling participants to collect follow-up data.
• Have remote options for recruitment and data collection where possible, in case participants are unable or unwilling to attend appointments. If visual assessment of patients is required, promote the use of video appointments but have back-up options available to suit patients and sites.

### Limitations

This research focuses on a single RCT within the UK, therefore may not be applicable to different locations, trial designs, disease areas or intervention types. Data obtained from trial sites was not always complete, depending on individual capacity to return trial data so may not be an accurate reflection of activity across all sites. In particular, approximately one third of sites responded to the survey and attended the site investigator meeting. As qualitative interviews were not undertaken, the robustness of the data may be limited. As the pandemic remains ongoing, we are unable to provide data on how effective the evaluated strategies are post-pandemic.
